# Supplementary material for: Developments and Performance of Artificial Intelligence Models Designed for Application in Endodontics: A Systematic Review
Source: Diagnostics (Basel). 2023 Jan 23;13(3):414. doi: 10.3390/diagnostics13030414 (PMC9913920; doi:10.3390/diagnostics13030414)
Supplement: Supplementary file 1 [file diagnostics-13-00414-s001.zip › diagnostics-2163898-supplementary.pdf]

diagnostics

Table S1: Assessment of risk of bias domains and applicability concerns.

		RISK OF BIAS				APPLICABILITY CONCERNS		
Serial No	Authors	Patient Selection	Index Test	Reference Standard	Flow and Timing	Patient Selection	Index Test	Reference Standard
1	Saghiri M. A. et al. [25]	Low	Low	Low	Low	High	Low	Low
2	Saghiri M. A et al. [26]	Low	Low	Low	Low	High	Low	Low
3	Kositbowornchai. S et al. [27]	Low	Low	Low	Low	High	Low	Low
4	Tumbelaka et al. [28]	High	Low	High	Unclear	High	Low	High
5	Johari. M et al. [29]	Low	Low	Low	Low	High	Low	Low
6	Shah H et al. [30]	Low	Low	Low	Low	Low	Low	Low
7	Ekert T et al. [31]	Low	Low	Low	Low	Low	Low	Low
8	Fukuda M et al. [32]	Low	Low	Low	Low	Low	Low	Low
9	Hiraiwa T et al. [33]	Low	Low	Low	Low	Low	Low	Low
10	Mallishery S et al. [34]	Low	Low	Low	Low	Low	Low	Low
11	Setzer FC et al. [35]	Low	Low	Low	Low	Low	Low	Low
12	Orhan K et al. [36]	Low	Low	Low	Low	Low	Low	Low
13	Endres MG et al. [37]	Low	Low	Low	Low	Low	Low	Low
14	Qiao X et al. [38]	Low	Low	Low	Low	High	Low	Low
15	Sherwood A A et al. [39]	Low	Low	Low	Low	Low	Low	Low
16	Li C W et al. [40]	Low	Low	Low	Low	Low	Low	Low
17	Vicory J et al. [41]	Low	Low	Low	Low	Low	Low	Low
18	Zheng L et al. [42]	Low	Low	Low	Low	Low	Low	Low
19	Moidu N P et al. [43]	Low	Low	Low	Low	Low	Low	Low
20	Pauwels R et al. [44]	Low	Low	Low	Low	Low	Low	Low
21	Jeon SJ et al. [45]	Low	Low	Low	Low	Low	Low	Low
22	Guo W et al. [45]	Low	Low	Low	Low	High	Low	Low
23	Lin X et al. [46]	Low	Low	Low	Low	Low	Low	Low
24	Gao X et al. [48]	High	Low	High	High	High	Low	High
25	Ngoc VTN et al. [49]	Low	Low	Low	Low	Low	Low	Low
26	Kirnbauer B et al. [50]	High	Low	High	Unclear	High	Low	High
27	Herbst CS et al. [51]	Low	Low	Low	Low	Low	Low	Low
28	Bayrakdar IS et al. [52]	High	Low	High	High	High	Low	High
29	Zhao L et al. [53]	Low	Low	Low	Low	Low	Low	Low
30	Hamdan MH et al. [54]	Low	Low	Low	Low	Low	Low	Low
31	Calazans MAA et al. [55]	Low	Low	Low	Low	Low	Low	Low
32	Yang S et al. [56]	Low	Low	Low	Low	Low	Low	Low
33	Xu T et al. [57]	Low	Low	Low	Low	Low	Low	Low
34	Qu Y et al. [58]	Low	Low	Low	Low	Low	Low	Low
35	Li Y et al. [59]	Low	Low	Low	Low	Low	Low	Low
36	Hu Z et al. [60]	Low	Low	Low	Low	Low	Low	Low
37	Vasdev D et al. [61]	Low	Low	Low	Low	Low	Low	Low
